# Correspondence

**Published:** 1896-06

**Authors:** 


### CORRESPONDENCE.

THE JOURNAL received the following letter from Dr. Cecil French, of 714 Twelfth Street, Washington, D. C., who is taking an active interest in this laudable undertaking, and we trust by giving it publicity in the JOURNAL that we may contribute to its successful attainment.

For some time the National Zoological Park has possessed in its collection a few of the various breeds of dogs, which have been kindly presented or loaned.

It is the intention of the officials, in order to provide object-lessons at the Capital for the people of the United States, to bring this department of the animal collection up to as complete a condition as possible. Accordingly an extensive and modern system of kennels is to be erected during the summer, so that every breed may be represented.

The appropriation voted by the House of Representatives for this institution cannot be devoted to the purchase of any animals; at the same time a clause prohibits selling of the same. Consequently the Park has to depend on gifts or loans of animals.

The officials of the Park have instructed me to extend to the leading kennel-breeders of the United States an invitation to coöperate in this movement, which is distinctly national in character, without attachment of any personal interest whatever. They desire that I request of you either the gift or loan of a representative of the breed in which you are interested. Acknowledgment of the same will in every case be made permanent by printed placard over each kennel, stating in bold letters the name and address of the donor or lender. The advantages that will accrue to the said donor or lender cannot be overestimated when regarded as an advertising medium, because the collection will take the form of a permanent exhibition to the many thousands of visitors from all parts of the country.

The officials will, of course, be gratified to receive a clear gift from you, but should you not see your way clear to this, will be very glad to receive a specimen as a permanent loan. In the case of a bitch, which would be preferred from an educational point of view, inasmuch as any young that might be bred would be interesting and instructive, the progeny would be considered the property of the lender, and would be returned or disposed of according to his directions.

In cases of sickness the best of medical attendance will be promptly given. The kennels will be in charge of competent attendants, and full information of the characteristics and points of the breed will be printed and attached to each kennel. All charges incidental to the removal and transportation of the animals will be borne by the Institution.

---

Dr. G. Leo Hagen Burger has received an appointment as milk-inspector for the Health Department of Brooklyn, N. Y.

